# Substrate recognition by the 4‐hydroxytryptamine kinase PsiK in psilocybin biosynthesis

**DOI:** 10.1002/1873-3468.15042

**Published:** 2024-10-24

**Authors:** Kai Rogge, Tobias Johannes Wagner, Dirk Hoffmeister, Bernhard Rupp, Sebastiaan Werten

**Affiliations:** ^1^ Institute of Pharmacy Friedrich Schiller University Jena Germany; ^2^ Research Group Pharmaceutical Microbiology, Leibniz Institute of Natural Product Research and Infection Biology Hans Knöll Institute Jena Germany; ^3^ Department of General, Inorganic and Theoretical Chemistry University of Innsbruck Austria; ^4^ k.‐k. Hofkristallamt San Diego CA USA

**Keywords:** enzyme, kinase, mutagenesis, natural product, phosphorylation, psilocybin, psychedelic, substrate recognition, X‐ray crystallography

## Abstract

Psilocybin, the natural hallucinogen from *Psilocybe* (magic) mushrooms, is a highly promising drug candidate for the treatment of depression and several other mental health conditions. Biosynthesis of psilocybin from the amino acid l‐tryptophan involves four strictly sequential modifications. The third of these, ATP‐dependent phosphorylation of the intermediate 4‐hydroxytryptamine, is catalysed by PsiK. Here we present a crystallographic analysis and a structure‐based mutagenesis study of this kinase, providing insight into its mode of substrate recognition. The results of our work will support future bioengineering efforts aimed at generating variants of psilocybin with enhanced therapeutic properties.

## Graphical abstract

X‐ray crystallography and structure‐guided mutagenesis were used to gain insight into substrate recognition by the kinase that enables biosynthesis of psilocybin, the hallucinogenic substance in *Psilocybe* (magic) mushrooms. The results of this work will support bioengineering efforts aimed at generating novel variants of psilocybin with improved therapeutic properties.

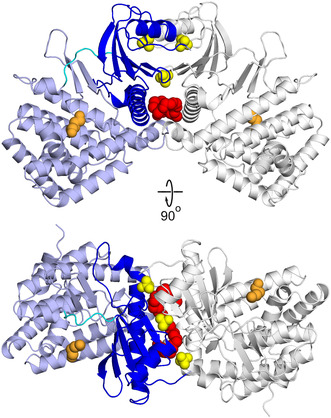

## Abbreviations

4‐HT, 4‐hydroxytryptamine

ASU, asymmetric unit

IPTG, isopropyl β‐d‐1‐thiogalactopyranoside

MME, monomethyl ether

MTR, methylthioribose

NCS, non‐crystallographic symmetry

PEG, polyethylene glycol

RMSD, root mean square deviation

**Fig. 1:**
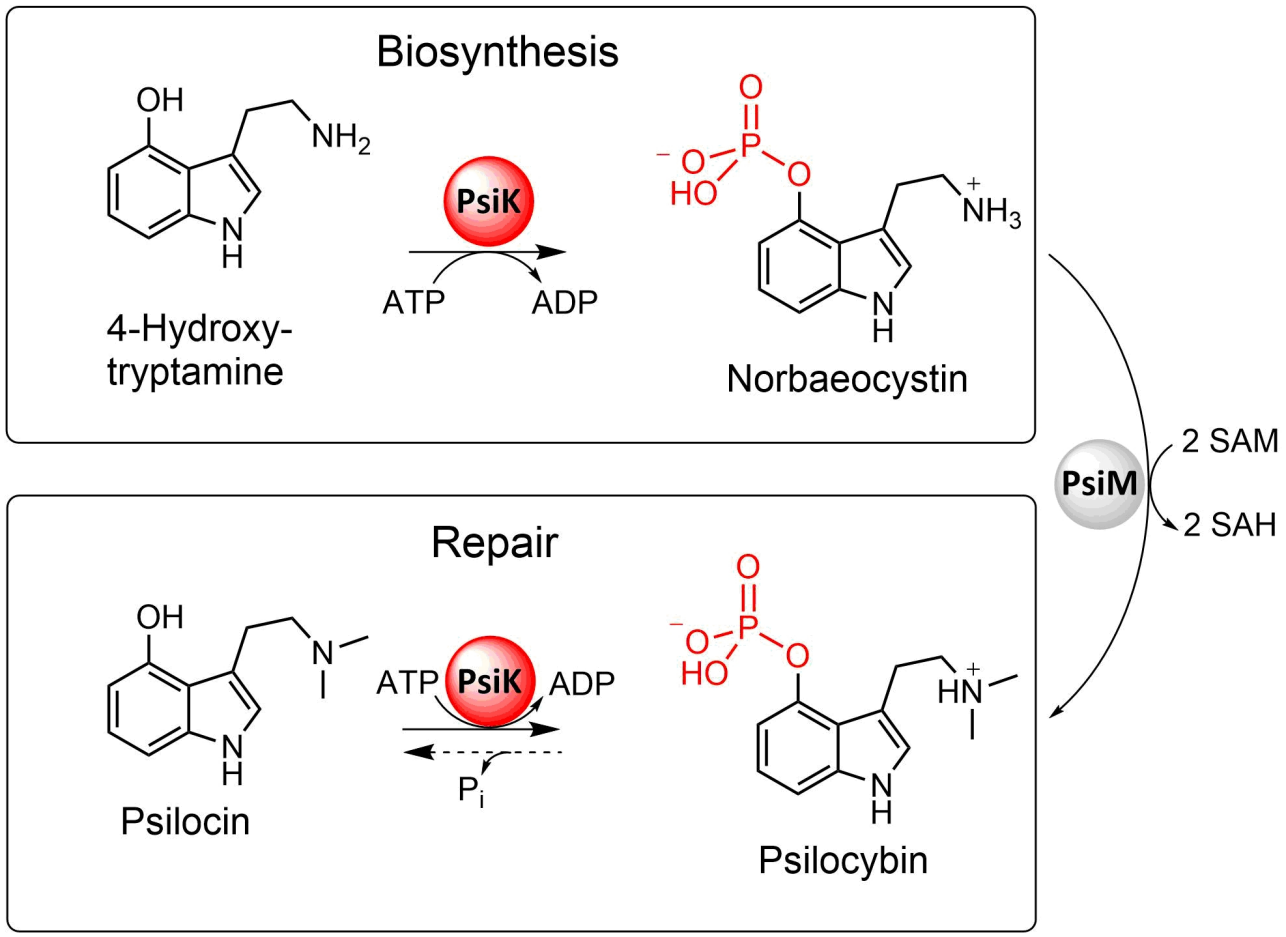
The double role of PsiK in *Psilocybe* mushrooms. PsiK uses the γ‐phosphate of ATP to phosphorylate 4‐hydroxytryptamine to norbaeocystin. The latter is the substrate of the final enzyme in the pathway, the dimethyltransferase PsiM. In addition, PsiK re‐phosphorylates the hydrolysis product psilocin, thereby restoring psilocybin.

Psilocybin (Fig. 1) is the main natural product of the *Psilocybe* genus and various other fungi, colloquially referred to as ‘magic mushrooms’ [1, 2, 3]. The phosphorylated indolethylamine is a chemically stable precursor of psilocin, a partial agonist of human 5‐hydroxytryptamine (5‐HT) receptors [4, 5, 6]. Clinical studies have demonstrated that treatment with psilocybin can markedly alleviate a variety of psychological conditions, such as major depressive disorder [7], substance dependence [8] and end‐of‐life anxiety [9]. Based on these findings, psilocybin has been attributed breakthrough therapy status by the US Food and Drug Administration, paving the way for accelerated approval.

The emerging interest in psilocybin as a therapeutic agent has led to numerous efforts to produce the compound more efficiently by means of biotechnological approaches and generate novel variants with improved pharmaceutical properties. In mushrooms, psilocybin is synthesised from the amino acid l‐tryptophan via a strictly sequential reaction pathway [10] that involves four dedicated enzymes: the gateway decarboxylase PsiD [11], the monooxygenase PsiH, the kinase PsiK [12, 13, 14] and the methyltransferase PsiM [15]. Genes encoding these enzymes have been identified in *Psilocybe cubensis* and several other fungi [10, 11, 14, 16, 17], enabling large‐scale heterologous production of psilocybin in microorganisms such as *Aspergillus nidulans* [18], *Escherichia coli* [19] and *Saccharomyces cerevisiae* [20]. Various analogues have also been generated, including ring‐methylated and halogenated variants [12, 21].

The penultimate reaction in the psilocybin biosynthetic pathway (Fig. 1) is catalysed by PsiK [10, 12, 13, 14], an ATP‐dependent enzyme that shows sequence homology to the eukaryotic protein kinase and kinase‐like domain superfamily. PsiK phosphorylates the l‐tryptophan‐derived intermediate 4‐hydroxytryptamine (4‐HT) to produce norbaeocystin. The ethylamine moiety of this compound is then dimethylated by the final enzyme in the pathway, PsiM, to produce psilocybin [15]. In addition to phosphorylating 4‐HT, PsiK also re‐phosphorylates any psilocin that forms *in vivo* due to spontaneous hydrolysis of psilocybin [14]. Psilocin is highly reactive and readily forms polymers that cause intracellular damage, for instance by precipitating proteins [22, 23]. Therefore, PsiK fulfils both a biosynthetic and a protective function.

Since the phosphorylation step in the organic synthesis of psilocybin remains challenging [24], PsiK is of particular biotechnological and pharmaceutical interest. In the present study, we have determined the X‐ray crystallographic structure of the kinase. Based on a detailed comparison to related enzymes we identify the likely binding site for 4‐HT and psilocin, which we confirm by means of site‐directed mutagenesis.

## Materials and methods

### Heterologous production and purification of PsiK for structural studies

*Escherichia coli* BL21 (DE3) was transformed with pJF44, an expression vector derived from pET‐28a (Merck KGaA, Darmstadt, Germany) that encodes full‐length *P. cubensis* PsiK (GenBank entry ASU62237.1) preceded by a hexahistidine tag and a recognition site for TEV protease. Cultures were grown at 37 °C in LB medium supplemented with 50 μg·mL^−1^ kanamycin and 1% glucose. At an OD_600nm_ of 0.5, isopropyl β‐d‐1‐thiogalactopyranoside (IPTG) was added to a final concentration of 1 mm, followed by protein expression for 5 h at 16 °C. Upon centrifugation (60 min at 10 000 ***g***, 4 °C), cells from 2 L of culture were resuspended in 30 mL buffer A (20 mm Tris/HCl pH 8.0, 400 mm NaCl) and disrupted by sonication on ice using a Sonopuls HD 2070 (Bandelin Electronic GmbH, Berlin, Germany). Insoluble material was removed by centrifugation for 1 h at 40 000 ***g*** and 4 °C. The lysate was then passed through a 0.45 μm filter (Whatman GmbH, Dassel, Germany) and applied to a 5 mL metal affinity chromatography Ni^2+^‐cartridge (GE Healthcare GmbH, Munich, Germany). After extensive washing, PsiK was eluted using a 0–500 mm imidazole gradient in buffer A. Peak fractions were pooled and dialysed for 12 h against 2 L buffer A at 4 °C to remove imidazol. This was followed by the addition of 0.5 mL TEV protease (1 mg·mL^−1^) and ADP to a final concentration of 0.25 mm. After a further 48 h at 4 °C, the solution was passed through the Ni^2+^‐cartridge again and PsiK eluted by means of a 0–100 mm imidazole gradient in buffer A. Selected fractions were pooled and ADP was added to a final concentration of 1 mm. The protein was further purified by gel filtration (Superdex 200, GE Healthcare GmbH) in a buffer containing 20 mm Tris/HCl pH 8.0, 400 mm NaCl and 1 mm DTT. Peak fractions were pooled and concentrated to 9.4 mg·mL^−1^ (0.20 mm) using a Vivaspin 20 ultrafiltration unit (10 kDa MWCO, Sartorius Lab Instruments GmbH, Göttingen, Germany). Protein concentration was determined spectroscopically, making use of a sequence‐based estimate of the molar absorption coefficient at 280 nm (47 650 m ^−1^·cm^−1^). Aliquots of the purified protein were flash‐frozen in liquid nitrogen and stored at −80 °C until further use.

### X‐ray crystallography

PsiK was crystallised at 4 °C using the sitting drop method. Drops of 0.4 μL were produced by mixing equal volumes of the protein solution and the reservoir buffer. The latter contained 100 mm HEPES pH 7.5, 2 m ammonium sulphate and 2% (w:v) PEG 550 MME. After several weeks, monoclinic crystals belonging to space group *C*2 began to appear.

Crystals were flash‐cooled in liquid nitrogen without the addition of further cryoprotectants. For X‐ray diffraction experiments, a temperature of 100 K was used. Data were collected at ESRF ID23‐2 [25] and processed using xds [26, 27]. Structures were solved by molecular replacement using phaser [28], in combination with the ccp4 suite [29]. A monomeric structure generated by alphafold [30, 31] was used as the search model. coot [32] and refmac [33, 34] were used for iterative rounds of model building and refinement. Data collection and refinement statistics can be found in Table 1.

**Table 1:** Data collection and structure refinement statistics for PsiK, PDB entry 9ETO. Values in parentheses pertain to the highest resolution shell.

Data collection	
X‐ray source	ESRF ID23‐2
Wavelength (Å)	0.8731
Detector	PILATUS3 X 2M
Space group	*C*2
Unit cell dimensions	
*a*, *b*, *c* (Å)	111.8, 70.14, 118.9
α, β, γ (°)	90, 95.84, 90
*V*_*m*_ (Å^3^·Da^−1^)	2.85
Solvent content (%)	56.9
Resolution range (Å)	43.1–2.54 (2.61–2.54)
Multiplicity	6.63 (6.85)
Completeness (%)	99.9 (99.9)
<*I*/σ(*I*)>	6.39 (0.91)
Wilson *B* (Å^2^)	60.6
*R*_merge_ (%)	27.5 (264.1)
*R*_meas_ (%)	29.9 (285.9)
*CC*_1/2_ (%)	99.1 (35.0)
Refinement	
Nr. reflections	
Used in refinement	28 916 (2126)
In test set	1483 (107)
*R*_work_	19.7 (44.0)
*R*_free_	24.3 (42.4)
Nr. protein residues	726
Nr. non‐hydrogen atoms	
Total	5797
Protein	5684
Other non‐solvent	101
Solvent	12
RMSD bond lengths (Å)	0.0064
RMSD bond angles (°)	1.7
Ramachandran	
Favoured (%)	96
Allowed (%)	4
Outliers (%)	0
Rotamer outliers (%)	6
Clash score	1.40
Average *B* (Å^2^)	62.9

All molecular graphics illustrations were produced with pymol 0.99rc6 (DeLano Scientific LLC, Palo Alto, CA, USA). Structural comparisons were performed using the SSM superposition method implemented in coot [32].

### Site‐directed mutagenesis

Mutagenesis of the *psiK* gene involved PCR amplification of the pET‐28a‐derived expression plasmid pJF23 [10]. Each gene variant was amplified in two fragments that overlapped in the area to be mutated, using the primers listed in Table S1. Thermal cycling conditions were: 98 °C for 30 s at the start; 35 cycles of 98 °C for 8 s, 61 °C for 20 s and 72 °C for 50 s; 72 °C for 5 min at the end. Phusion DNA polymerase (New England Biolabs GmbH, Frankfurt, Germany) was used according to the manufacturer's instructions. The dNTPs were added to 200 μm and oligonucleotide primers to 0.5 μm final concentration. Oligonucleotide oKR102 was used as the forward primer to amplify the upstream fragments, while oKR103 served as the reverse primer for the downstream fragments.

The 5′‐end of all upstream fragments and the 3′‐end of all downstream fragments overlapped with the multiple cloning site of vector pET‐28a, which was linearised by *Bam*HI and *Hind*III restriction. Plasmids were assembled with the NEBuilder HiFi DNA Assembly Kit (New England Biolabs GmbH). The resulting expression plasmids, pKR20‐pKR23 and pTW04‐pTW07, correspond to PsiK variants W319A, W226A, D249A, E251A, F319A, Y264A, L252A and L184A, respectively.

### Heterologous production and purification of PsiK for enzymatic assays

To produce hexahistidine‐tagged PsiK wild type and variants, *E. coli* KRX (Promega GmbH, Walldorf, Germany) was transformed with plasmid pJF23 (for PsiK wild type) or the expression plasmids mentioned above. For each transformant, a 5 mL culture was grown overnight at 37 °C in LB medium supplemented with 50 μg·mL^−1^ kanamycin and then used to inoculate 500 mL of 2 × YT medium with 50 μg·mL^−1^ kanamycin. This culture was incubated at 37 °C to an OD_600nm_ of 0.6. After induction with l‐rhamnose to a final concentration of 0.1%, the cultures were shaken for another 20 h at 16 °C. The biomass was collected by centrifugation at 4000 ***g*** and 4 °C, resuspended in 7.5 mL lysis buffer (50 mm phosphate buffer, 500 mm NaCl, 20 mm imidazole, pH 8) and lysed with a Bandelin Sonopuls GM 3200 sonicator. To remove cell debris, the lysate was centrifuged (13 751 ***g***, 20 min, 4 °C). The cleared supernatant was passed through a 0.45 μm filter (Whatman) and purified using a 1 mL HisTrap HP column (Cytiva Europe GmbH, Munich, Germany). Following an extensive wash, PsiK was eluted using a 0–250 mm imidazole gradient in lysis buffer. Purification was verified by SDS/PAGE (Fig. S1), and the protein was quantified photometrically at a wavelength of 280 nm. For use in subsequent activity assays, the enzyme was rebuffered on an Amicon column (Merck KGaA) and eluted with 50 mm phosphate buffer (pH 7.5) for subsequent activity assays.

### Enzyme activity assays

Unless stated otherwise, kinase activity assays were set up in triplicates in a total volume of 50 μL. All reactions contained 1 mm MgCl_2_, 1 mm ATP and 0.5 mm substrate (either 4‐HT or psilocin). Reactions were started by adding PsiK (0.1 μm final concentration) and incubated at 40 °C. After 4 min, reactions were stopped by flash‐cooling in liquid nitrogen. The material was then lyophilised, resuspended in 50 μL methanol and placed in an ultrasonic bath for 3 min, followed by centrifugation.

Chromatographic analysis was performed on an Agilent Infinity II UHPLC–MS instrument, equipped with a 6130 single quadrupole mass detector, and by electrospray ionisation in positive mode, and a Phenomenex Luna Omega polar C_18_ column (50 × 2.1 mm, 1.6 μm particle size). Solvent A was 0.1% (v/v) formic acid in water, solvent B was acetonitrile. To detect tryptamines, the following gradient was applied: 0 min, 1% B; within 3 min to 5% B; within another minute to 100% B; held at 100% B for 2 min. The flow rate was 0.5 mL·min^−1^.

## Results

### The crystal structure of PsiK

**Fig. 2:**
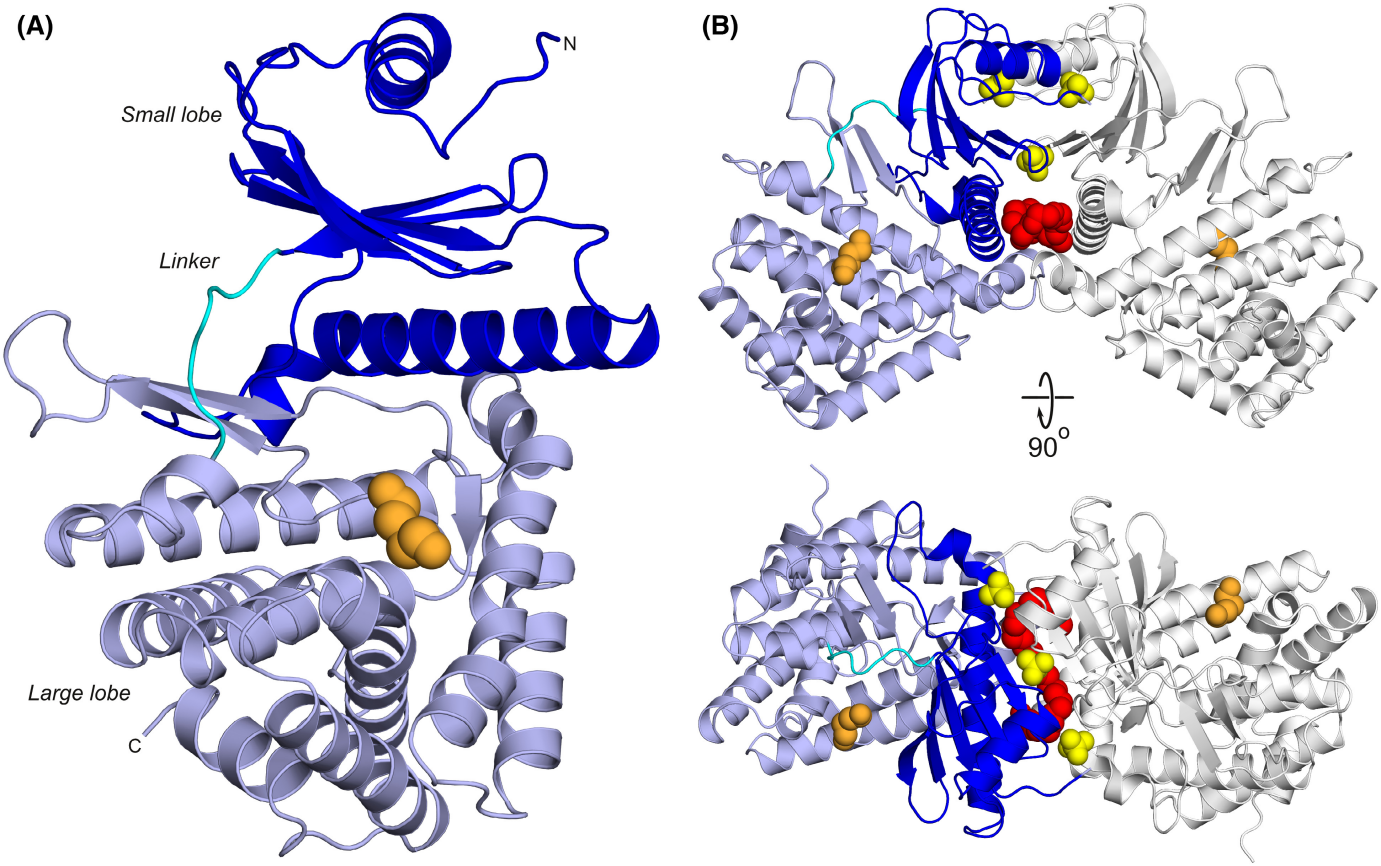
The crystal structure of PsiK. (A) The bilobed protein kinase fold of PsiK (chain A). The large and small lobes are shown as light and dark blue ribbon diagrams, respectively, with the region that links the two (amino acids 119–125) in cyan. A short PEG fragment that occupies the putative substrate‐binding site is represented by a space‐filling model (orange). (B) The asymmetric unit (ASU) of the PsiK crystal, with chains A and B shown as ribbon diagrams (colours for chain A as in panel A, chain B grey). A PEG 550 MME chain and three sulphate ions trapped between the two protein molecules are represented by space‐filling models (red and yellow, respectively). Short PEG fragments that occupy the putative substrate‐binding sites are shown in orange.

The X‐ray structure of PsiK, crystallised in the absence of any substrate, was determined at a resolution of 2.5 Å (Fig. 2, Table 1). Consistent with sequence‐based predictions, the enzyme adopts the typical bilobed structure found in eukaryotic protein kinases (Fig. 2A). The small N‐terminal lobe (comprising residues 1–118) consists of a five‐stranded antiparallel β‐sheet and two α‐helices with αβββαββ topology, while the larger C‐terminal lobe (residues 126–362) is almost entirely α‐helical. The N‐ and C‐terminal lobes are connected by a short linker (residues 119–125).

The two PsiK molecules in the asymmetric unit of the crystal (ASU) are related by 2‐fold non‐crystallographic symmetry (NCS) and can be superimposed with a C_α_‐RMSD of 0.13 Å for 363 matched atoms. Intriguingly, the A and B chains appear to form a homodimer, mainly through contacts between the N‐terminal lobes (Fig. 2B). This mode of association is unusual and does not resemble protein–protein interactions previously observed in known homodimeric kinases. The interaction between PsiK molecules is mediated to a large extent by a polyethylene glycol 550 monomethyl ether (PEG 550 MME) molecule and three sulphate ions from the crystallisation buffer, which are sequestered at the interface between the neighbouring protein chains. Analysis using the PDBePISA server [35] suggests that the homodimeric assembly is not stable in the absence of PEG 550 MME and sulphate, with a negative Δ*G* ^diss^ of −6.2 kcal·mol^−1^. It therefore seems likely that the dimerisation of PsiK in our structure is a crystallisation artefact. This is consistent with the results of gel filtration experiments, which indicate that PsiK exists as a monomer in solution [14].

### PsiK closely resembles methylthioribose kinases

**Fig. 3:**
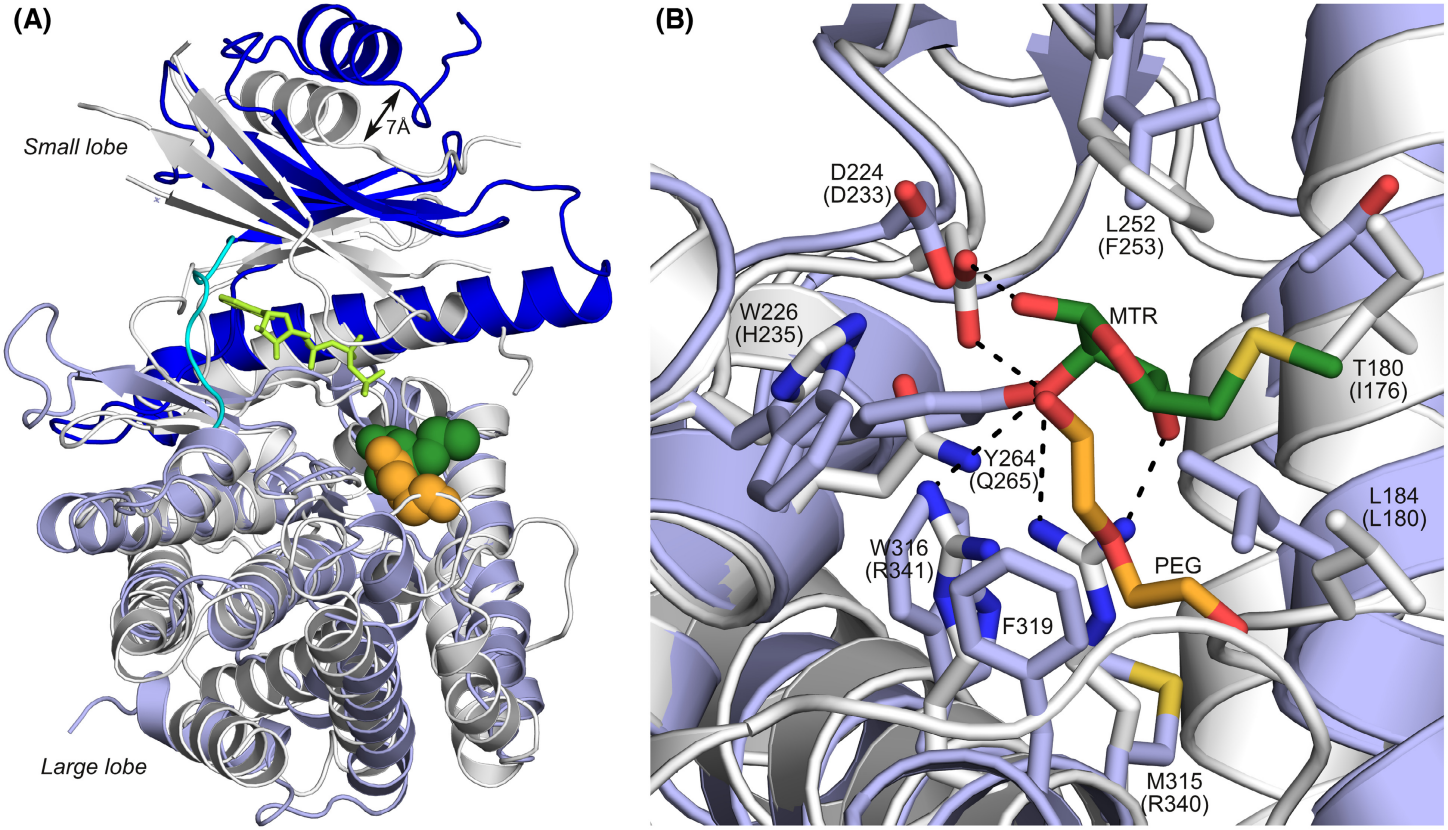
Comparison of PsiK and the MTR kinase of *B. subtilis*. (A) Superposition of PsiK (colours as in Fig. 2A) and the MTR kinase of *B. subtilis* (grey). The non‐hydrolysable ATP analogue (phosphomethylphosphonic acid adenylate ester, or AppCp) in the MTR kinase structure is shown as a bright green stick model. The PEG fragment in the putative substrate binding site of PsiK and the MTR molecule bound to the MTR kinase are shown as space‐filling models (orange and dark green, respectively). A double arrow indicates the 7 Å shift of the small lobe of PsiK with respect to the MTR kinase, leading to an open conformation that is reminiscent of the inactive state of eukaryotic protein kinases. (B) Detailed view of the putative substrate‐binding pocket of PsiK, showing structurally equivalent residues in the MTR kinase of *B. subtilis* (labels of the latter in brackets). Ligands are shown as stick models. Key hydrogen bonds in the structure of the MTR kinase are represented by dashed lines. Phe319 of PsiK does not have an equivalent in the MTR kinase, but is in direct van der Waals contact with the PEG molecule.

Dali [36, 37] analysis identified the methylthioribose (MTR) kinases from *Bacillus subtilis* (PDB code 2PUN, see Fig. 3A) and *Arabidopsis thaliana* (PDB code 2PYW) as the most similar PDB entries [38, 39]. The Dali *Z*‐scores for these structures are 27.0 and 26.6, respectively, with C_α_‐RMSD values of 3.1 and 3.2 Å (307 superimposed atoms). PsiK shares several structural features with the MTR kinases that were previously identified as distinguishing the latter from true protein kinases [38]. Like the MTR kinases, PsiK lacks the characteristic DFG motif typically found at the beginning of the activation loop of protein kinases. Moreover, an atypical DXE motif (residues 249–251, equivalent to residues 250–252 in the MTR kinase of *B. subtilis*) is likely to bind the catalytically important Mg^2+^‐ion that interacts simultaneously with the β‐ and γ‐phosphates of ATP. In sharp contrast to PsiK and the majority of protein kinases, however, the two MTR kinases are known to form stable homodimers in solution [38, 39].

Superposition of PsiK and the MTR kinase of *B. subtilis* reveals that in the former the small and large lobes are separated by an additional ~ 7 Å (Fig. 3A). Thus, PsiK adopts an ‘open’ nucleotide‐free conformation, reminiscent of the inactive conformation of regulatory protein kinases in eukaryotes [40, 41]. This observation is somewhat surprising, as the more closely related MTR kinases have been proposed to exclusively exist in the active (or ‘closed’) conformation, even in the absence of nucleotides and substrates [38]. It remains possible, however, that the open conformation seen in PsiK is imposed by crystal packing forces.

### Substrate binding and specificity

The MTR kinase of *B. subtilis* mainly recognises its substrate by means of an acidic residue (Asp233), a twin arginine motif (Arg340, Arg341) and the so‐called W‐loop (residues 64–79) [38]. Superposition of the crystal structures of the MTR kinase and PsiK shows that Asp233 in the former corresponds to Asp224 in the latter (Fig. 3B). This suggests that the negatively charged side chain of Asp224 in PsiK likewise participates in substrate binding, for instance by recognising the protonated ethylamine moiety of 4‐HT and psilocin. In apparent agreement with this hypothesis, a D224A mutant of PsiK was found to be entirely inactive [14]. The twin arginine motif of the *B. subtilis* MTR kinase is not conserved in PsiK, but appears to be structurally equivalent to a pair of solvent‐exposed hydrophobic residues (Met315 and Trp316) that could interact with the indole ring of the substrate. Together with Thr180, Leu184, Trp226, Leu252 and Phe319, these residues form a hydrophobic pocket that in our X‐ray structure sequesters part of a PEG molecule from the crystallisation buffer (Figs 2 and 3). The W‐loop of the *B. subtilis* MTR kinase does not have an equivalent in PsiK. Instead, the corresponding residues in the protein sequence extend helix α2, which in PsiK is several turns longer than in the MTR kinases.

**Fig. 4:**
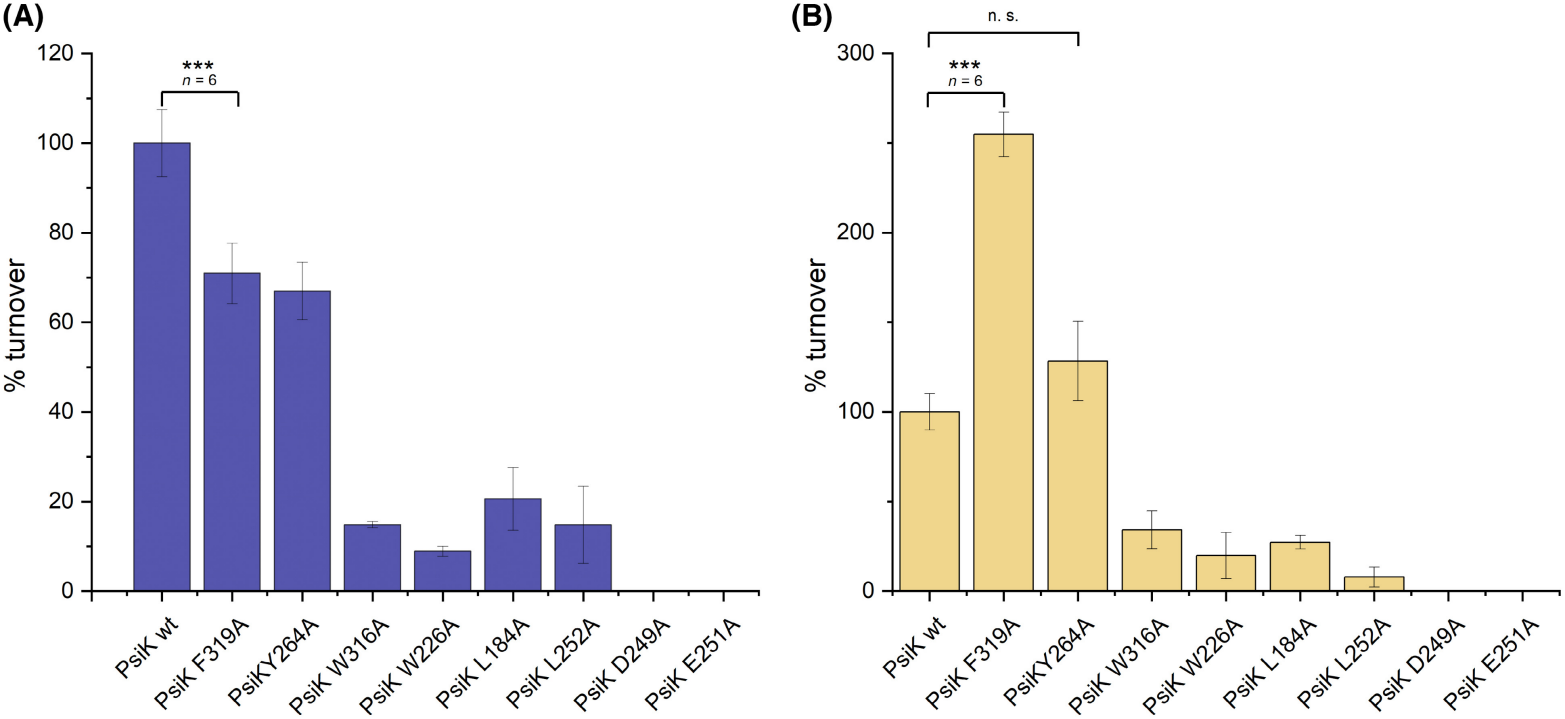
Mutational analysis of the putative substrate‐binding pocket in PsiK. For each substrate, phosphorylation by PsiK variants is shown as a percentage of turnover by PsiK^wt^. Unless stated otherwise, *n* = 3. The significance of selected differences was analysed by means of a *t*‐test (***: *P* = 0.001, n.s.: not significant). Error bars correspond to standard deviations. (A) Phosphorylation of 4‐HT to norbaeocystin. (B) Phosphorylation of psilocin to psilocybin.

In order to confirm the location of the putative substrate‐binding site in PsiK we produced six alanine mutants, targeting hydrophobic side chains that we predicted to play a role in ligand recognition (F319A, Y264A, W316A, W226A, L184A and L252A). The acidic residues of the DXE motif, expected to interact with the catalytically important Mg^2+^‐ion, were mutated to alanine in two further variants (D249A and E251A). The activity of each mutant was measured in two independent *in vitro* assays, one with the biosynthetic precursor 4‐HT and the other with the hydrolysis product psilocin (Fig. 4A,B, respectively). Irrespective of the substrate, the D249A and E251A variants were completely inactive, reflecting the critical role of the DXE motif in positioning the catalytic Mg^2+^‐ion. All of the other substitutions also affected phosphorylation of the 4‐HT substrate, particularly W316A, W226A, L184A and L252A. The negative effect of the F319A and Y264A substitutions was smaller but still statistically significant. Thus, all of the hydrophobic residues that we mutated are likely to play a role in substrate recognition.

For most variants, activity towards psilocin mirrored the results from the 4‐HT assay. In contrast, PsiK^Y264A^ was found to be fully active when phosphorylating psilocin, while PsiK^F319A^ showed a significant increase (2.5‐fold, with *n* = 6 and *P* = 0.001) in turnover compared to PsiK^wt^. These substrate‐dependent effects may indicate that residues Y264 and F319 are in direct contact with the ethylamine moiety, an interaction likely to be affected by the presence of the methyl groups in psilocin.

## Discussion

In the present study, we have elucidated the crystal structure and substrate‐binding mode of the 4‐hydroxytryptamine kinase PsiK from *P. cubensis*, the enzyme that catalyses the phosphorylation step in the biosynthetic pathway generating psilocybin from l‐tryptophan [10]. Our results show that PsiK is structurally unrelated to most of the small molecule kinases that act in primary metabolism, such as those belonging to the hexokinase, ribokinase and galactokinase families [42]. Like MTR kinases [38, 39], PsiK instead shows a strong resemblance to eukaryotic protein kinases and is likely to be evolutionarily related to these. Of note, a recent study indicated that another enzyme from the psilocybin biosynthetic pathway, the methyltransferase PsiM, arose from the METTL16 family of epitranscriptomic writer proteins [15]. Thus, ancient regulatory factors appear to have been at the origin of a major part of the psilocybin pathway.

PsiK not only catalyses an essential step in the biosynthesis of psilocybin from l‐tryptophan, but in addition mediates a salvage reaction that restores psilocybin following its spontaneous hydrolysis to psilocin (Fig. 1). Conflicting evolutionary pressures may have resulted in a compromise, that is, a kinase capable of processing both 4‐HT and psilocin at the expense of some of the activity towards either of these substrates. Intriguingly, we found that the F319A mutation in PsiK led to an increase in activity with respect to psilocin, whereas activity towards 4‐HT was negatively affected. This indicates that the salvage reaction was not fully optimised in the course of evolution, apparently because maintaining maximal efficiency of the 4‐HT phosphorylation reaction was more important for *Psilocybe* fitness than an increase in the activity towards psilocin. Consistent with this idea, 4‐HT appears to be the more deleterious of the two compounds, as it is much more rapidly oxidised than psilocin [43].

Our crystallographic model of PsiK provides a structural basis for targeted engineering of the kinase, particularly with the aim of accommodating modified substrates. Although Speeter‐Anthony chemistry provides a convenient and well‐established way to produce large amounts of natural as well as modified tryptamines, the phosphorylation step required to generate psilocybin and its derivatives remains more challenging [24, 44]. *In vivo* methods based on recombinant microorganisms carrying the *psi* genes circumvent this problem [18, 19, 20], but are less flexible in the sense that generating analogues of psilocybin requires multiple enzymes to accept non‐natural substrates [21]. If PsiK can be adapted to accommodate substituted tryptamines, a hybrid approach combining Speeter‐Anthony chemistry to generate tryptamines with kinase‐mediated *in vitro* phosphorylation is likely to prove both efficient and flexible.

## Author contributions

B.R. and D.H. initiated the project and obtained funding; S.W., K.R. and T.J.W. produced protein samples; S.W. obtained crystals, collected X‐ray diffraction data and solved the PsiK structure; K.R. and T.J.W. generated mutants and performed enzymatic assays, supervised by S.W. and D.H.; S.W. coordinated the project and wrote the manuscript, with contributions from D.H., K.R. and B.R.

### Peer review

The peer review history for this article is available at https://www.webofscience.com/api/gateway/wos/peer‐review/10.1002/1873‐3468.15042.

## Supporting information


**Fig. S1.** Coomassie‐stained SDS/PAGE of purified PsiK variants.
**Table S1.** Oligonucleotide primers used for site‐directed mutagenesis.

## Data Availability

The crystallographic structure of PsiK and the corresponding X‐ray diffraction data have been deposited in the PDB with accession number 9ETO.
