# Supplementary material for: Substrate recognition by the 4‐hydroxytryptamine kinase PsiK in psilocybin biosynthesis
Source: FEBS Lett. 2024 Oct 24;599(3):447–55. doi: 10.1002/1873-3468.15042 (PMC11808438; doi:10.1002/1873-3468.15042)

Supplementary Table S1

Oligonucleotide primers used for site-directed mutagenesis.

Name	Sequence (5´→ 3´)	PsiK variant	Direction
oKR93	GTGATGGCGACCGACTTTATGC	W316A	For
oKR94	GCATAAAGTCGGTCGCCATCAC		Rev
oKR95	CGGACCTGGCGAGTGGAAATAT	W226A	For
oKR96	ATATTTCCACTCGCCAGGTCCG		Rev
oKR97	ATATATCCTGGCCTGGGAACTTTGC	D249A	For
oKR98	GCAAAGTTCCCAGGCCAGGATATAT		Rev
oKR99	CCTGGATTGGGCACTTTGCAAG	E251A	For
oKR100	CTTGCAAAGTGCCCAATCCAGG		Rev
oKR102	GCAAATGGGTCGCGGATCCATGGCGTTCGATCTC	Primers for 5‘- and 3‘- terminus of *psiK*	For
oKR103	AGTGCGGCCGCAAGCTTTTACGCAGTGGATGATTCC		Rev
oTW3	CCTGGGCGCTTTCTTGGGT	Y264A	For
oTW4	ACCCAAGAAAGCGCCCAGG		Rev
oTW5	GTGGACCGACGCTATGCAGT	F319A	For
oTW6	ACTGCATAGCGTCGGTCCAC		Rev
oTW7	CTTCAGACCAGGCGTATCAAACC	L184A	For
oTW8	GGTTTGATACGCCTGGTCTGAAG		Rev
oTW9	ATTGGGAAGCTTGCAAGTACGG	L252A	For
oTW10	CCGTACTTGCAAGCTTCCCAAT		Rev

Supplementary Fig. S1

Coomassie-stained SDS-PAGE of purified PsiK variants.

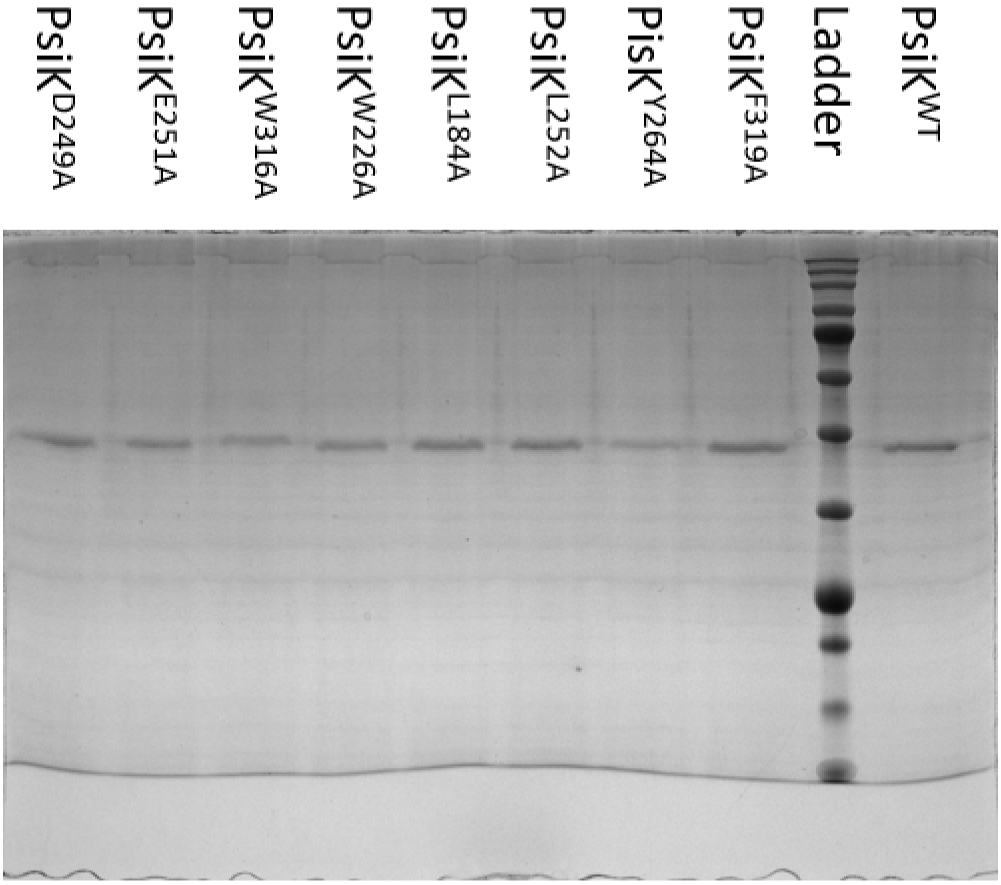

Supplement: Supplementary file 1 — Fig. S1. Coomassie‐stained SDS/PAGE of purified PsiK variants. Table S1. Oligonucleotide primers used for site‐directed mutagenesis. [file FEB2-599-447-s001.pdf]
